# Sex‐Stratified Association of Serum Uric Acid With Multisite Bone Mineral Density Among Inpatients With Type 2 Diabetes: A Cross‐Sectional Study

**DOI:** 10.1155/jdr/4367218

**Published:** 2026-08-12

**Authors:** Shangjian Li, Weihong Lu, Xiumei Luo, Kai Wang, Jingqi Zhou, Silan Zheng, Ning Chen

**Affiliations:** ^1^ Department of Endocrinology, Zhongshan Hospital (Xiamen), Fudan University, Xiamen, China, fudan.edu.cn; ^2^ Department of Gynaecology, Zhongshan Hospital (Xiamen), Fudan University, Xiamen, China, fudan.edu.cn

**Keywords:** bone mineral density, cross-sectional study, serum uric acid, Type 2 diabetes mellitus

## Abstract

**Objective:**

This study sought to investigate the correlation between serum uric acid (SUA) levels and bone mineral density (BMD) among patients with Type 2 diabetes mellitus (T2DM).

**Methods:**

A single‐center retrospective cross‐sectional analysis was performed. A total of 249 hospitalized patients with T2DM (147 men and 102 postmenopausal women) were recruited from Zhongshan Hospital (Xiamen), Fudan University, between June 2024 and June 2025. Baseline clinical characteristics were first compared between patients with normal BMD and those with osteopenia or osteoporosis. All participants were subsequently stratified into a normal uric acid (NUA) group and a hyperuricemia (HUA) group based on SUA concentrations. BMD measurements at the femoral neck, total hip, and lumbar spine were compared across the two groups. Multivariate logistic regression analysis was applied to determine independent factors associated with the risk of osteopenia and osteoporosis.

**Results:**

Of the 249 enrolled participants, 178 (71.5%) were diagnosed with osteopenia or osteoporosis. Relative to patients with normal BMD, individuals with osteopenia/osteoporosis were older, had a higher proportion of female cases, exhibited elevated osteocalcin (OCN) levels and increased fracture risk, and presented significantly lower body mass index (BMI), estradiol (E2), and SUA levels. In the overall cohort, as well as in male and postmenopausal female subgroups, higher SUA levels were significantly and positively correlated with higher BMD at all three skeletal sites (all *p* < 0.05). Multivariate logistic regression demonstrated that OCN served as an independent risk factor for osteopenia and osteoporosis, whereas BMI, E2, and SUA served as independent protective factors against these bone disorders (all *p* < 0.05).

**Conclusion:**

Elevated SUA levels are correlated with higher BMD and a reduced risk of osteopenia and osteoporosis in T2DM patients. BMI, E2, OCN, and SUA act as independent predictors of bone status in this patient population. Routine SUA testing may facilitate the identification of T2DM patients at high risk of osteopenia and osteoporosis.

## 1. Introduction

Alongside urbanization, population aging and dietary transitions in China, the prevalence of overweight, obesity and Type 2 diabetes mellitus (T2DM) has risen sharply. National epidemiological surveys have indicated that the prevalence of diabetes among Chinese adults aged 18 years and older reached 11.2%, with T2DM accounting for over 90% of all diabetic cases [1]. Meanwhile, metabolic disorders represented by hyperuricemia (HUA) have become increasingly prevalent. Among hospitalized T2DM patients in China, the comorbidity rate of T2DM and HUA exceeded 22%, leading to complex metabolic interactions between the two conditions [2]. Accumulating clinical evidence confirms that HUA is closely associated with chronic kidney disease, hypertension, nonalcoholic fatty liver disease, dyslipidemia, and cardiovascular and cerebrovascular events. Furthermore, HUA exacerbates insulin resistance and accelerates the progression of T2DM [3].

Osteoporosis is a well‐recognized chronic complication of T2DM. Persistent hyperglycemia, insulin resistance, dysregulated bone turnover, and chronic low‐grade inflammation contribute to reduced bone mass, impaired bone microarchitecture, and a marked increase in fracture risk. Epidemiological data show that the prevalence of osteopenia and osteoporosis ranges from 65% to 75% among hospitalized T2DM patients, and postmenopausal women and elderly men face a higher risk of fragility fractures [4]. Insulin resistance constitutes a key shared pathological mechanism underlying both T2DM and HUA: Insulin resistance reduces renal uric acid excretion and elevates SUA levels, and in turn, increased SUA further aggravates insulin resistance, forming a vicious cycle [5].

The role of SUA in bone metabolism remains controversial—a phenomenon widely known as the “uric acid redox paradox” in existing research. As the terminal metabolite of human purine metabolism, circulating SUA functions as an endogenous antioxidant that scavenges reactive oxygen species (ROS), alleviates oxidative damage to osteoblasts and osteoclasts, and maintains the homeostasis of bone remodeling [6, 7]. Multiple observational and genetic studies have verified that moderately elevated SUA is correlated with higher BMD and a lower risk of osteoporosis in the elderly, postmenopausal women, and diabetic patients [8, 9]. Nevertheless, excessive intracellular accumulation of SUA activates nicotinamide adenine dinucleotide phosphate oxidase, triggering massive production of ROS and proinflammatory mediators, including tumor necrosis factor‐*α* and interleukin‐1*β*, and further activating the NLRP3 inflammasome. In addition, SUA overaccumulation inhibits vitamin D synthesis and induces secondary hyperparathyroidism, ultimately accelerating bone resorption and bone loss [10–12]. Several European cohort studies have reported that excessively high SUA increases the risk of hip fractures (HFs) in middle‐aged and older adults, particularly males. Studies focusing on adolescent females have also demonstrated the adverse impacts of elevated SUA on skeletal health [13, 14].

To date, research findings regarding the association between SUA and BMD in T2DM patients remain inconsistent. Such discrepancies are primarily attributed to variations in study populations, age stratification, gender distribution, SUA cut‐off values, renal function status, and adjustments for confounding factors. Most existing studies lack detailed stratification by sex and menopausal status, as well as comprehensive analyses incorporating multiple bone turnover markers. Given the high comorbidity of T2DM, HUA, and osteoporosis in clinical practice, clarifying the relationship between SUA and BMD in T2DM patients is of great clinical significance for the integrated management of multiple metabolic disorders and early identification of patients at high fracture risk. Accordingly, we conducted this single‐center retrospective cross‐sectional study to analyze the correlations between SUA and BMD at the femoral neck, total hip, and lumbar spine, as well as the independent influencing factors for osteopenia and osteoporosis. The findings aim to provide observational evidence for the comprehensive assessment of skeletal health in T2DM patients.

## 2. Materials and Methods

### 2.1. Study Population

**Figure 1:**
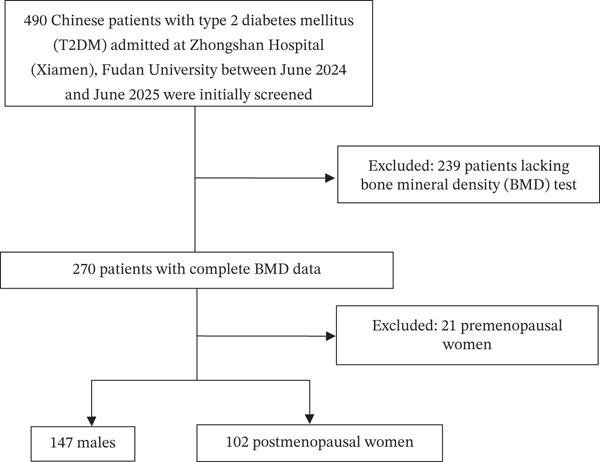
Study population screening flowchart.

This single‐center retrospective cross‐sectional study enrolled 249 hospitalized patients with T2DM (147 males and 102 postmenopausal females) from the Department of Endocrinology, Zhongshan Hospital (Xiamen), Fudan University, between June 2024 and June 2025 (Figure 1). All participants met the 1999 World Health Organization (WHO) diagnostic criteria for diabetes mellitus and tested negative for glutamic acid decarboxylase (GAD) antibodies. In accordance with the 2019 Primary Care Guidelines for Gout and Hyperuricemia issued by the Chinese Medical Association, HUA was defined as a serum uric acid level greater than 420 *μ*mol/L detected on two nonconsecutive days, regardless of sex.

Diagnoses of osteoporosis were established based on dual‐energy X‐ray absorptiometry (DXA) results in line with the 1994 WHO criteria: Normal BMD was defined as a *T*‐score within ±1 standard deviation (SD) of the peak bone mass of sex‐ and ethnicity‐matched healthy adults; osteopenia was defined as a *T*‐score between −1.0 and −2.5 SD; osteoporosis was defined as a *T* − score ≤ −2.5 SD. Severe osteoporosis was diagnosed when osteoporosis was accompanied by one or more fractures.

All participants were first divided into a normal BMD group and an osteopenia/osteoporosis group for baseline characteristic comparisons. Subsequently, all patients were categorized into the NUA group and HUA group according to SUA levels to compare BMD across the three skeletal sites. Multivariate logistic regression analysis was performed among all 249 participants to screen for independent factors associated with osteopenia and osteoporosis, with normal bone mass set as the reference group and osteopenia/osteoporosis defined as the positive outcome.

#### 2.1.1. Exclusion Criteria

Exclusion criteria include the following: (1) patients with Type 1 diabetes, gestational diabetes, or other secondary diabetes; (2) patients with mental disorders who were unable to complete physical examinations and data collection; (3) patients with active infectious diseases or acute inflammatory stress; (4) patients with severe dysfunction of major organs including the heart, liver, and kidneys; (5) patients who had taken medications affecting uric acid production, transport, or metabolism within the preceding 2 months; (6) patients with malignant tumors or other diseases causing secondary elevation of SUA; (7) patients who had used drugs interfering with bone metabolism, such as glucocorticoids, vitamin D, calcium (Ca) supplements, and bisphosphonates prior to enrollment; and (8) patients with endocrine disorders (thyroid, parathyroid, adrenal, and gonadal diseases) or gastrointestinal or renal diseases affecting Ca and phosphorus (P) metabolism, autoimmune diseases, or congenital and acquired bone metabolic disorders.

This study was approved by the Ethics Committee of Zhongshan Hospital (Xiamen), Fudan University (Approval No. B2024‐093). Written informed consent was obtained from all participants and their family members.

### 2.2. Data Collection and Laboratory Measurements

#### 2.2.1. General Data Collection

Demographic information, medical history, smoking, and drinking status were recorded. Height, body weight, waist circumference, hip circumference, and blood pressure were measured, and body mass index (BMI) was calculated. The duration of diabetes and diabetic complications were also documented.

#### 2.2.2. Biochemical and Bone Metabolic Indicators

Venous blood samples were collected after an overnight fast of no less than 8 h. The detected indicators included fasting plasma glucose (FPG), triglyceride (TG), total cholesterol (TC), high‐density lipoprotein cholesterol (HDL‐c), low‐density lipoprotein cholesterol (LDL‐c), glycated hemoglobin (HbA1c), alanine transaminase (ALT), aspartate transaminase (AST), blood urea nitrogen (BUN), serum creatinine (CREA), estimated glomerular filtration rate (eGFR), SUA, serum Ca, serum P, parathyroid hormone (PTH), osteocalcin (OCN), N‐terminal propeptide of Type I procollagen (PINP), and *β*‐C‐terminal telopeptide of Type I collagen (CTX‐*β*).

Serum lipid profiles were measured using an automatic biochemical analyzer; FPG was detected via the glucose oxidase method; HbA1c was determined by high‐performance liquid chromatography. The homeostatic model assessment for insulin resistance (HOMA‐IR) was calculated accordingly. Levels of 25‐hydroxyvitamin D_3_ [25(OH)D], PTH, OCN, and PINP were quantified using an automatic electrochemiluminescence analyzer (Cobas e602, Roche Diagnostics).

#### 2.2.3. BMD Measurement and Fracture Risk Assessment

BMD at the lumbar spine, femoral neck, and total hip was measured using a DXA scanner (QDR4500A, Hologic Inc., Waltham, M**assachusetts**, U**nited** S**tates**). The 10‐year risks of major osteoporotic fracture (MOF) and HF were evaluated using the Chinese version of the Fracture Risk Assessment Tool (FRAX). Daily quality control was implemented for the DXA device, and all acquired data were double‐checked by two independent researchers. Cases with missing key data were excluded from the final analysis.

### 2.3. Calculation Formula

HOMA−IR=FPG mmol/L×fasting insulin μU/mL/22.5.

### 2.4. Statistical Analysis

Sporadic minor missing data were handled with pairwise deletion, while samples with severe missing key variables were excluded. The Shapiro–Wilk test was used for normality testing. Normally distributed continuous variables were presented as mean ± SD and compared using the independent samples *t*‐test. Nonnormally distributed continuous variables were expressed as median (interquartile range [IQR]) and analyzed using nonparametric tests. Categorical variables were reported as frequencies and percentages and compared using the chi‐square test.

Multivariate logistic regression was constructed using the backward elimination method, and variables with *p* < 0.05 were retained in the final regression model. All statistical analyses were performed using SPSS 25.0 software. A two‐tailed *p* value < 0.05 was considered statistically significant.

## 3. Results

### 3.1. Comparison of Baseline Characteristics Between the Normal BMD Group and the Osteopenia/Osteoporosis Group

A total of 249 T2DM patients were enrolled in this study, among whom 178 (71.5%) were diagnosed with osteopenia or osteoporosis. Compared with patients with normal BMD, those with osteopenia/osteoporosis were older and had a higher proportion of female participants. They also presented significantly lower height, body weight, BMI, waist circumference, hip circumference, serum estradiol (E2), and SUA levels, as well as markedly higher OCN levels and 10‐year fracture risks assessed by FRAX (all *p* < 0.05). Stratified analysis by sex revealed consistent trends in both male and postmenopausal female subgroups: Patients with reduced BMD had lower BMI, E2, and SUA, alongside higher long‐term fracture risk. No significant intergroup differences were observed for other biochemical, glycolipid, and hepatorenal indicators (all *p* > 0.05) (Tables 1 and 2).

**Table 1:** Comparison of basic data between normal BMD and osteoporosis/osteopenia groups in all T2DM patients.

	Normal BMD group, *n* = 71	Osteoporosis/osteopenia group, *n* = 178	*p* value
Age (years)	55.39 ± 12.51	58.74 ± 11.3	0.042
Gender, *n* (%)			0.026
Male	50 (70.4)	98 (55.1)	
Female	21 (29.6)	80 (44.9)	
Height (cm)	167.78 ± 9.09	163.42 ± 9.08	0.001
Weight (kg)	73.81 ± 12.66	65.56 ± 12.05	< 0.001
BMI (kg/m^2^)	26.31 ± 4.92	24.48 ± 3.75	0.002
Waist circumference (cm)	91.28 ± 10.01	87.60 ± 9.34	0.008
Hip circumference (cm)	96.61 ± 7.06	93.06 ± 6.79	0.001
Systolic blood pressure (mmHg)	130.28 ± 16.79	131.29 ± 16.48	0.666
Diastolic blood pressure (mmHg)	81.89 ± 10.81	82.0 ± 9.58	0.936
Diabetes duration (years)	6.59 ± 6.67	8.31 ± 7.33	0.088
Diabetic retinopathy, *n* (%)	22 (31.0)	51 (28.7)	0.715
Diabetic nephropathy, *n* (%)	13 (18.3)	28 (15.7)	0.620
Diabetic peripheral neuropathy, *n* (%)	9 (12.7)	27 (15.2)	0.614
Diabetic peripheral vascular disease, *n* (%)	47 (66.2)	123 (69.1)	0.657
The percentage of patients on insulin therapy, *n* (%)	23 (32.4)	56 (31.5)	0.886
The percentage of patients on GLP‐1RA, *n* (%)	21 (29.6)	48 (27.0)	0.633
ALT (U/L)	36.87 ± 84.32	24.92 ± 18.57	0.240
AST (U/L)	25.1 ± 40.07	20.64 ± 11.79	0.359
BUN (mmol/L)	5.0 (4.33, 6.18)	5.55 (4.6, 6.7)	0.069
CREA (*μ*mol/L)	81.77 ± 46.43	73.32 ± 23.30	0.147
eGFR (mL/min/1.73 m^2^)	91.14 ± 22.91	90.09 ± 19.37	0.715
UA (*μ*mol/L)	396.83 ± 93.16	340 ± 99.43	0.033
TC (mmol/L)	4.50 ± 1.10	4.59 ± 1.70	0.708
TG (mmol/L)	1.72 ± 0.89	2.09 ± 2.44	0.221
HDL‐c (mmol/L)	1.11 (0.91, 1.27)	1.07 (0.89, 1.32)	0.998
LDL‐c (mmol/L)	2.65 ± 0.98	2.51 ± 1.01	0.293
FPG (mmol/L)	8.81 ± 3.38	8.12 ± 2.92	0.126
HbA1c (%)	9.33 ± 2.51	9.06 ± 2.23	0.400
HOMA‐IR	3.72 ± 3.79	4.82 ± 7.62	0.418
Blood calcium (mmol/L)	2.31 ± 0.11	2.32 ± 0.27	0.516
Blood phosphorus (mmol/L)	1.21 ± 0.18	1.24 ± 0.18	0.146
PTH (pg/mL)	40.22 ± 25.77	34.85 ± 12.45	0.097
OCN (ng/mL)	13.17 ± 4.64	14.86 ± 5.86	0.031
PINP (ng/mL)	0.41 ± 0.22	0.45 ± 0.23	0.404
CTX‐*β* (ng/mL)	47.47 ± 28.93	45.98 ± 18.79	0.690
25(OH)2D3 (nmol/L)	56.96 ± 28.32	60.64 ± 21.10	0.324
E2 (pmol/L)	96.50 ± 52.39	63.47 ± 44.87	< 0.001
LH (mIU/mL)	16.20 ± 14.98	18.35 ± 14.71	0.301
FSH (mIU/mL)	24.89 ± 27.98	31.81 ± 28.40	0.083
P (nmol/L)	0.45 ± 0.36	0.40 ± 0.26	0.312
*T* (nmol/L)	11.01 ± 8.09	8.16 ± 8.05	0.013
PRL (mIU/mL)	333.70 ± 152.02	426.25 ± 888.09	0.384
FRAX MOF score	1.73 ± 0.54	3.43 ± 2.08	< 0.001
FRAX HF score	0.19 ± 0.17	1.21 ± 1.35	< 0.001

Abbreviations: 25(OH)2D3, 25‐hydroxyvitamin D3; ALT, alanine aminotransferase; AST, aspartate aminotransferase; BMI, body mass index; BUN, blood urea nitrogen; CREA, blood creatinine; CTX‐*β*, Type I collagen carboxytelopeptide beta cross‐linked peptide; E2, estradiol; eGFR, calculated glomerular filtration rate; FPG, fasting plasma glucose; FRAX HF, Fracture Risk Assessment Tool probability of hip fracture; FRAX MOF, Fracture Risk Assessment Tool probability of major osteoporotic fracture; FSH, follicle‐stimulating hormone; HbA1c, glycosylated hemoglobin; HDL‐c, high‐density lipoprotein cholesterol; HOMA‐IR, homeostasis model assessment for insulin resistance; LDL‐c, low‐density lipoprotein cholesterol; LH, luteinizing hormone; OCN, osteocalcin; P, progesterone; PINP, Type I procollagen aminotelopeptide; PRL, prolactin; PTH, parathyroid hormone; T, testosterone; TC, total cholesterol; TG, triglyceride; UA, blood uric acid.

**Table 2:** Comparison of basic data between normal BMD and osteoporosis/osteopenia groups in men and postmenopausal women T2DM patients.

	Men			Postmenopausal women		
	Normal BMD group, *n* = 50	Osteoporosis/osteopenia group, *n* = 98	*p*value	Normal BMD group, *n* = 21	Osteoporosis/osteopenia group, *n* = 80	*p* value
Age (years)	52.88 ± 12.99	54.32 ± 11.91	0.502	61.38 ± 9.03	64.16 ± 7.61	0.155
Height (cm)	171.67 ± 5.88	169.53 ± 5.70	0.034	158.52 ± 8.75	155.83 ± 6.36	0.116
Weight (kg)	76.66 ± 12.12	70.84 ± 11.31	0.004	67.04 ± 59.02	59.02 ± 9.50	0.001
BMI (kg/m^2^)	25.97 ± 3.54	24.58 ± 3.24	0.018	27.13 ± 7.27	24.36 ± 4.32	0.028
Waist circumference (cm)	93.03 ± 9.34	89.52 ± 8.61	0.026	86.50 ± 10.46	85.24 ± 9.71	0.628
Hip circumference (cm)	96.89 ± 6.90	93.83 ± 6.31	0.009	95.86 ± 7.62	92.13 ± 7.26	0.055
Systolic blood pressure (mmHg)	130.08 ± 17.01	128.71 ± 16.10	0.633	130.76 ± 16.67	134.44 ± 16.50	0.367
Diastolic blood pressure (mmHg)	82.70 ± 10.71	83.48 ± 9.16	0.645	79.95 ± 11.07	80.19 ± 9.84	0.925
Diabetes duration (years)	5.81 ± 6.45	6.32 ± 5.85	0.632	8.43 ± 6.95	10.75 ± 8.22	0.239
Diabetic retinopathy, *n* (%)	15 (30.0)	23 (23.5)	0.390	7 (33.0)	28 (35.0)	0.886
Diabetic nephropathy, *n* (%)	10 (20.0)	16 (16.3)	0.579	3 (14.3)	12 (15.0)	0.935
Diabetic peripheral neuropathy, *n* (%)	5 (10.0)	7 (7.1)	0.547	5 (19.0)	20 (25.0)	0.568
Diabetic peripheral vascular disease, *n* (%)	33 (66.0)	66 (67.3)	0.867	14 (66.7)	57 (71.3)	0.682
The percentage of patients on insulin therapy, *n* (%)	15 (30.0)	30 (29.8)	0.939	8 (38.1)	26 (32.5)	0.629
The percentage of patients on GLP‐1RA, *n* (%)	14 (28.0)	32 (32.7)	0.765	7 (33.3)	16 (20.0)	0.817
ALT (U/L)	20.5 (14, 37.5)	20 (15, 27)	0.522	20.48 ± 9.33	24.51 ± 20.57	0.967
AST (U/L)	18 (14, 25)	17 (14, 22)	0.607	20.48 ± 9.33	21.71 ± 13.93	0.702
BUN (mmol/L)	5.92 ± 3.07	5.75 ± 1.81	0.671	5.24 ± 2.06	6.18 ± 2.24	0.086
CREA (*μ*mol/L)	91.74 ± 51.19	79.65 ± 22.17	0.116	58.05 ± 16.93	65.65 ± 22.43	0.151
eGFR (mL/min/1.73 m^2^)	89.90 ± 25.00	93.67 ± 18.49	0.302	94.10 ± 17.11	85.75 ± 19.62	0.078
UA (*μ*mol/L)	398.12 ± 92.44	342.52 ± 94.69	0.022	393.76 ± 93.57	337.04 ± 103.77	0.016
TC (mmol/L)	4.50 ± 1.03	4.41 ± 1.12	0.616	4.51 ± 1.29	4.81 ± 2.20	0.558
TG (mmol/L)	1.77 ± 0.97	2.29 ± 3.14	0.250	1.62 ± 0.67	1.84 ± 1.05	0.077
HDL‐c (mmol/L)	1.04 ± 0.23	1.05 ± 0.29	0.862	1.24 (1.01, 1.31)	1.14 (0.96, 1.37)	0.849
LDL‐c (mmol/L)	2.69 ± 0.94	2.48 ± 0.91	0.192	2.57 ± 1.10	2.54 ± 1.12	0.900
FPG (mmol/L)	8.68 ± 3.41	8.50 ± 3.14	0.760	9.14 ± 3.38	7.64 ± 2.58	0.092
HbA1c (%)	9.26 ± 2.51	9.15 ± 2.33	0.786	9.50 ± 2.57	8.95 ± 2.09	0.310
HOMA‐IR	2.82 (1.34, 4.31)	2.51 (1.49, 3.92)	0.951	5.07 ± 5.79	5.25 ± 9.92	0.939
Blood calcium (mmol/L)	2.30 ± 0.12	2.31 ± 0.82	0.470	2.32 ± 0.81	2.33 ± 0.13	0.574
Blood phosphorus (mmol/L)	1.16 ± 0.16	1.21 ± 0.19	0.119	1.31 ± 0.18	1.28 ± 0.18	0.541
PTH (pg/mL)	36 (27.65, 43.23)	34.1 (26.1, 42.8)	0.284	46.47 ± 11.17	34.67 ± 12.38	0.547
OCN (ng/mL)	11.97 ± 3.70	12.80 ± 3.68	0.197	16.03 ± 5.43	17.35 ± 6.97	0.423
PINP (ng/mL)	44.04 ± 30.47	42.63 ± 16.59	0.761	55.61 ± 23.55	50.05 ± 20.52	0.286
CTX‐*β* (ng/mL)	0.40 ± 0.23	0.43 ± 0.19	0.515	0.45 ± 0.21	0.47 ± 0.28	0.804
25(OH)2D3 (nmol/L)	49.4 (38.68, 74.95)	60.4 (43.1, 71.7)	0.222	54.80 ± 23.39	60.19 ± 16.52	0.228
E2 (pmol/L)	117.80 ± 32.23	93.45 ± 37.64	< 0.001	22.8 (18.35, 42.3)	18.35 (17.1, 30.63)	0.027
LH (mIU/mL)	9.80 ± 4.74	8.21 ± 3.89	0.054	31.43 ± 19.60	30.77 ± 13.52	0.856
FSH (mIU/mL)	11.48 ± 9.17	10.23 ± 6.11	0.390	56.84 ± 31.82	58.23 ± 21.85	0.815
P (nmol/L)	0.45 ± 0.30	0.41 ± 0.26	0.371	0.44 ± 0.50	0.39 ± 0.27	0.530
T (nmol/L)	15.38 ± 5.24	14.44 ± 5.46	0.314	0.59 ± 0.33	0.48 ± 0.38	0.203
PRL (mIU/mL)	287.4 (206.2, 402.8)	267.2 (205.6, 420.0)	0.521	344.93 ± 134.20	451.46 ± 1090.10	0.657
FRAX MOF score	1.50 ± 0.35	2.54 ± 1.29	< 0.001	2.4 (1.8, 2.7)	3.8 (3.1, 4.91)	< 0.001
FRAX HF score	0.15 (0.1, 0.2)	0.7 (0.3, 1.3)	< 0.001	0.2 (0.1, 0.3)	0.95 (0.5, 1.75)	< 0.001

### 3.2. Comparison of BMD Between the NUA Group and the HUA Group

After stratification by SUA levels, BMD values at the femoral neck, total hip, and lumbar spine in the HUA group were significantly higher than those in the NUA group across the overall cohort, male subgroup, and postmenopausal female subgroup (all *p* < 0.05), indicating that BMD increased with elevated SUA concentrations (Tables 3 and 4).

**Table 3:** Comparison of BMD between the NUA group and the UA group in all T2DM patients.

	*U* *A* ≤ 420 *μ* mol/L, *n* = 199	*U* *A* > 420 *μ* mol/L, *n* = 50	*p* value
Femoral neck BMD	0.68 ± 0.12	0.76 ± 0.12	0.037
Hip BMD	0.82 ± 0.13	0.91 ± 0.15	0.014
Lumbar spine BMD	0.86 ± 0.15	0.99 ± 0.14	0.033

**Table 4:** Comparison of BMD between the NUA group and the UA group in men and postmenopausal women T2DM patients.

	Men			Postmenopausal women		
	*U* *A* ≤ 420 *μ* mol/L, *n* = 113	*U* *A* > 420 *μ* mol/L, *n* = 34	*p* value	*U* *A* ≤ 420 *μ* mol/L, *n* = 86	*U* *A* > 420 *μ* mol/L, *n* = 16	*p* value
Femoral neck BMD	0.72 ± 0.13	0.79 ± 0.10	0.024	0.64 ± 0.13	0.70 ± 0.12	0.031
Hip BMD	0.87 ± 0.11	0.95 ± 0.12	0.032	0.75 ± 0.14	0.84 ± 0.11	0.028
Lumbar spine BMD	0.90 ± 0.12	1.02 ± 0.11	0.046	0.83 ± 0.16	0.92 ± 0.13	0.021

### 3.3. Multivariate Logistic Regression Analysis for Osteopenia/Osteoporosis

Six sequential logistic regression models were established using the backward elimination approach. The final optimal model retained four independent influencing factors: BMI, E2, OCN, and SUA (all *p* < 0.05). After adjusting for confounding factors, BMI, E2, and SUA were identified as independent protective factors against osteopenia and osteoporosis, with higher levels corresponding to a lower risk of bone loss. OCN was confirmed as an independent risk factor, and elevated OCN levels increased the risk of osteopenia and osteoporosis. Age, sex, diabetes duration, and eGFR showed no independent correlation with bone loss (all *p* > 0.05) (Table 5).

**Table 5:** Multivariate logistic regression analysis of independent risk factors for osteoporosis/osteopenia in all T2DM patients.

Variables	Beta value	*p*value	95% CI
Model 1			
Age (years)	1.038	0.994–1.084	0.092
Gender	0.669	0.204–2.187	0.506
BMI (kg/m^2^)	0.893	0.800–0.997	0.045
Diabetes duration (years)	1.011	0.959–1.065	0.692
HbA1c (%)	0.951	0.802–1.128	0.564
E2 (pmol/L)	0.983	0.970–0.997	0.016
eGFR (mL/min/1.73 m^2^)	1.001	0.978–1.025	0.932
OCN (ng/mL)	1.075	1.003–1.153	0.042
UA (*μ*mol/L)	0.997	0.982–1.011	0.036
Model 2			
Age (years)	1.037	0.997–1.079	0.068
Gender	0.672	0.207–2.183	0.509
BMI (kg/m^2^)	0.893	0.801–0.997	0.044
Diabetes duration (years)	1.010	0.959–1.064	0.698
HbA1c (%)	0.952	0.803–1.128	0.568
E2 (pmol/L)	0.983	0.970–0.997	0.016
OCN (ng/mL)	1.075	1.003–1.152	0.041
UA (*μ*mol/L)	0.997	0.982–1.010	0.037
Model 3			
Age (years)	1.039	1.001–1.079	0.062
Gender	0.693	0.216–2.222	0.538
BMI (kg/m^2^)	0.893	0.800–0.998	0.045
HbA1c (%)	0.951	0.803–1.126	0.558
E2 (pmol/L)	0.983	0.970–0.997	0.015
OCN (ng/mL)	1.073	1.003–1.148	0.042
UA (*μ*mol/L)	0.998	0.984–1.009	0.038
Model 4			
Age (years)	1.042	1.002–1.079	0.060
Gender	0.683	0.214–2.181	0.520
BMI (kg/m^2^)	0.891	0.798–0.994	0.039
E2 (pmol/L)	0.983	0.970–0.997	0.015
OCN (ng/mL)	1.074	1.004–1.149	0.039
UA (*μ*mol/L)	0.997	0.985–1.008	0.037
Model 5			
Age (years)	1.039	1.002–1.077	0.057
BMI (kg/m^2^)	0.886	0.795–0.988	0.030
E2 (pmol/L)	0.986	0.977–0.996	0.004
OCN (ng/mL)	1.067	1.001–1.138	0.047
UA (*μ*mol/L)	0.997	0.989–1.007	0.036
Model 6			
BMI (kg/m^2^)	0.889	0.799–0.990	0.032
E2 (pmol/L)	0.984	0.974–0.993	0.001
OCN (ng/mL)	1.068	1.002–1.139	0.048
UA (*μ*mol/L)	0.998	0.990–1.007	0.036

## 4. Discussion

### 4.1. Main Findings and Clinical Implications

With global population aging, the comorbidity of T2DM and osteoporosis has become a major public health concern in China. Patients with T2DM carry a substantially higher risk of fragility fractures compared with the general population [15]. In the present study, the overall prevalence of osteopenia and osteoporosis among hospitalized T2DM patients reached 71.5%, with a prevalence of 65.1% in males and 79.4% in postmenopausal females. These results are consistent with recent epidemiological data on bone metabolic disorders among Chinese T2DM patients [16].

Univariate analyses demonstrated that T2DM patients with osteopenia/osteoporosis were older, more likely to be postmenopausal females, and had lower BMI, E2, and SUA levels, as well as higher OCN levels and 10‐year fracture risk. Sex‐stratified analyses validated the above trends in both males and postmenopausal females. Comparisons between the NUA and HUA groups confirmed that higher SUA was correlated with elevated BMD at all detected skeletal sites. Multivariate logistic regression further verified that BMI, E2, and SUA were independently negatively associated with the risk of osteopenia and osteoporosis, while OCN was independently positively associated with this risk.

BMI is widely recognized as a protective factor for skeletal health. Higher body weight increases mechanical loading on bones, which stimulates bone formation and mitigates bone loss [17]. As a critical sex hormone, E2 inhibits osteoclast differentiation and bone resorption; the decline in E2 after menopause is the primary cause of rapid bone loss in elderly women [18, 19]. OCN is a specific biomarker of bone formation. Elevated OCN indicates excessive bone turnover in T2DM patients, which disrupts the balance between bone formation and resorption and consequently increases the risk of osteopenia and osteoporosis.

### 4.2. Mechanisms Underlying the Association Between SUA and BMD in T2DM

The interaction between SUA and BMD in T2DM patients is complex and regulated by multiple metabolic and inflammatory mediators. Inconsistent conclusions from previous studies are essentially attributed to the dual redox properties of SUA [12]. The bone‐protective effect of moderately elevated SUA observed in this study can be explained by its antioxidant capacity. Oxidative stress is a core pathogenic mechanism underlying diabetic osteoporosis. Long‐term hyperglycemia leads to excessive ROS accumulation, which impairs osteoblast activity, promotes osteoclast proliferation, and accelerates bone loss [10]. As a major endogenous antioxidant in plasma, SUA scavenges free radicals, relieves oxidative damage to bone cells, and stabilizes bone microarchitecture, thereby exerting protective effects on bone tissue [20, 21]. Multiple Mendelian randomization studies conducted in East Asian populations have also confirmed that genetically determined higher SUA is linked to a lower risk of osteoporosis, which supports our present findings [22]. Notably, this correlation does not confirm a direct causal protective effect of SUA on bone metabolism. The dual effects of SUA cannot be overlooked. Excessive intracellular SUA accumulation activates oxidative pathways, induces NLRP3 inflammasome–related inflammatory responses, inhibits vitamin D synthesis, and triggers secondary hyperparathyroidism, ultimately exacerbating bone loss [11]. This may explain why several cohort studies have reported that elevated SUA is associated with increased fracture risk. We speculate that the effects of SUA on bone metabolism follow a concentration threshold: Moderately increased SUA exerts antioxidant and bone‐protective effects, while persistently excessive SUA shifts to a pro‐oxidative and proinflammatory state and impairs skeletal health. Renal function is an important confounding factor affecting both uric acid metabolism and bone metabolism. Uric acid is mainly excreted by the kidneys; reduced eGFR leads to uric acid retention, and renal dysfunction also causes disorders of Ca–P metabolism and impaired vitamin D activation, which indirectly affect BMD [3]. Although eGFR was included in the baseline indicators, this study did not perform stratification by renal function, which is one of its limitations.

### 4.3. Comparison With Previous Literature and Analysis of Heterogeneity

Numerous studies have explored the relationship between SUA and BMD, yet relevant conclusions remain inconsistent. A 6‐year longitudinal study of Chinese T2DM patients reported a positive correlation between SUA and BMD, which is consistent with our results [8]. Retrospective studies focusing on postmenopausal women also verified that higher SUA correlates with better skeletal status [23, 24]. In contrast, European studies found that elevated SUA increases HF risk in middle‐aged and elderly individuals, and studies in adolescents have indicated the detrimental effects of high SUA on bone health [13, 14].

Four major factors account for these heterogeneous findings. First, differences in study populations (e.g., age, sex, ethnicity, menopausal status, and underlying diseases) alter the correlation pattern between SUA and BMD. Second, the threshold effect of uric acid redox activity leads to contradictory conclusions when different SUA cut‐off values are adopted. Third, early studies failed to fully adjust for confounding factors such as renal function and medications related to uric acid and bone metabolism. Fourth, variations in study design exist: Cross‐sectional studies only reflect correlations, whereas longitudinal and interventional studies better capture dynamic changes of biomarkers. The present study strictly excluded patients using medications interfering with uric acid and bone metabolism and conducted stratification analyses by sex and menopausal status, which improves the clinical applicability of our conclusions in the T2DM population.

### 4.4. Clinical Implications

This cross‐sectional study confirmed that elevated SUA is correlated with higher BMD and a lower risk of osteopenia and osteoporosis among hospitalized T2DM patients. Combined with BMI, E2, and OCN, SUA can serve as an auxiliary biomarker for evaluating bone status in T2DM patients. Routine SUA detection in T2DM patients helps clinicians comprehensively identify individuals at high risk of abnormal bone mass and formulate individualized skeletal health management strategies. It should be emphasized that this study cannot establish causal relationships, and its findings cannot be used as a basis for adjusting clinical urate‐lowering therapy. HUA is an independent risk factor for cardiac and renal complications in T2DM patients. Urate‐lowering regimens should be formulated comprehensively based on SUA levels, renal function, cardiovascular status, and gout history. For T2DM patients complicated with both HUA and osteoporosis, clinicians need to balance the risks of cardiac/renal damage caused by HUA and bone loss and implement individualized management. The hypothesis that urate‐lowering treatment may attenuate the bone‐protective effects of SUA is merely a speculation based on cross‐sectional data, which requires further verification via large‐sample prospective cohort studies and clinical intervention trials.

### 4.5. Strengths and Limitations of the Study

#### 4.5.1. Strengths

First, this study focused on patients with the triple comorbidity of T2DM, HUA, and osteoporosis. BMD at three classic skeletal sites and multiple bone turnover markers were detected to comprehensively characterize bone metabolism. Second, strict inclusion and exclusion criteria were applied to exclude patients with secondary bone diseases and those taking medications interfering with uric acid and bone metabolism, reducing the confounding effects of exogenous drugs on the results. Third, stratification analyses were performed in male and postmenopausal female subgroups, clarifying the characteristics of the SUA–BMD association across different sex groups [19].

#### 4.5.2. Limitations

As a cross‐sectional study, this work cannot determine causal associations, and reverse causality cannot be ruled out. Second, all participants were inpatients recruited from a single medical center; thus, the conclusions cannot be generalized to community‐dwelling and outpatient T2DM populations. In addition, only postmenopausal women were enrolled, so the results are not applicable to premenopausal female T2DM patients [25]. Third, several key confounding factors closely related to bone metabolism, such as physical activity and dietary patterns, were not fully collected. Fourth, the relatively small sample size (249 cases) limits the robustness of the conclusions.

#### 4.5.3. Prospects for Future Research

We propose the following directions for subsequent research: (1) Conduct large‐scale, multicenter prospective cohort studies to clarify the causal relationship between SUA and BMD in T2DM patients, (2) establish nondiabetic control groups to compare differences in the SUA–bone metabolism association between diabetic patients and the general population, (3) combine the uric acid/creatinine ratio and inflammatory factors to explore the intermediate mechanisms by which SUA regulates bone metabolism, and (4) carry out clinical intervention trials to observe changes in BMD and bone turnover markers before and after standardized urate‐lowering therapy in T2DM patients, so as to provide high‐level evidence for clinical medication management.

## Author Contributions

S.L., W.L., and N.C. wrote the main manuscript text. X.L., K.W., J.Z., and S.Z. contributed to data collection and data cleaning. All authors reviewed the final manuscript. S.L. and W.L. contributed equally to this work.

## Funding

No funding was received for this manuscript.

## Ethics Statement

This study was approved by the Human Research Ethics Committee of Zhongshan Hospital (Xiamen, China), Fudan University (No. B2024‐093). The study was conducted in accordance with the principles of good clinical practice, the Declaration of Helsinki, and the International Conference on Harmonization Good Clinical Practice guidelines. All participants provided written informed consent.

## Conflicts of Interest

The authors declare no conflicts of interest.

## Data Availability

The data that support the findings of this study are available from the corresponding author, upon reasonable request.
